# Perillartine

**DOI:** 10.1107/S1600536809031225

**Published:** 2009-08-15

**Authors:** Xian-You Yuan, Min Zhang, Seik Weng Ng

**Affiliations:** a Department of Biology and Chemistry, Hunan University of Science and Engineering, Yongzhou, Hunan 425100, People’s Republic of China; b Department of Chemistry, University of Malaya, 50603 Kuala Lumpur, Malaysia

## Abstract

The chiral title compound [systematic name: 4-(1-methyl­vinyl)cyclo­hexene-1-carbaldehyde oxime], C_10_H_15_NO, crystallizes with two mol­ecules in the asymmetric unit, one of which shows disorder of its propenyl substituent over two sets of sites in a 50:50 ratio. In both mol­ecules, the six-membered carbaldehyde oxime ring adopts an approximate envelope conformation in which the C atom bearing the propenyl substituent represents the flap position. In both mol­ecules, the plane passing through the propenyl substituent is nearly perpendicular to the mean plane of the six-membered ring [dihedral angles = 84.6 (6) and 87.4 (3)°]. In the crystal, the two independent mol­ecules are linked by a pair O—H⋯N hydrogen bonds across a pseudo-inversion centre, generating a dimer. The unit cell of the known racemate of the title compound is similar to the cell found here, but with space group *P* 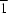.

## Related literature

Perillartine or perillartin [(*S*)-4-(prop-1-en-2-yl)cyclo­hex-1-ene carbaldehyde oxime], the oxime of perillaldehyde, is 2000 times sweeter than sucrose; see the handbook of artificial sweeteners by O’Brien Nabors & Gelardi (2001 ▶). For the crystal structure of the racemic compound, see: Hooft *et al.* (1990 ▶).
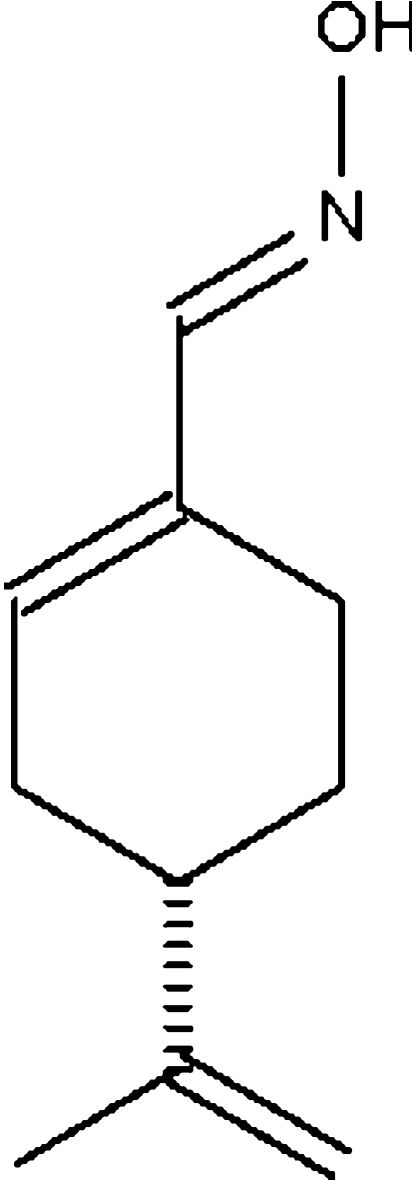


## Experimental

### 

#### Crystal data

C_10_H_15_NO
*M* *_r_* = 165.23
Triclinic, 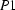

*a* = 7.2679 (6) Å
*b* = 8.1702 (7) Å
*c* = 8.9426 (8) Å
α = 105.150 (1)°
β = 95.658 (1)°
γ = 104.602 (1)°
*V* = 488.25 (7) Å^3^
*Z* = 2
Mo *K*α radiation
μ = 0.07 mm^−1^
*T* = 293 K
0.48 × 0.42 × 0.22 mm

#### Data collection

Bruker SMART diffractometer
Absorption correction: none
4074 measured reflections
2078 independent reflections
1457 reflections with *I* > 2σ(*I*)
*R*_int_ = 0.010

#### Refinement

*R*[*F* ^2^ > 2σ(*F* ^2^)] = 0.043
*wR*(*F* ^2^) = 0.139
*S* = 1.07
2078 reflections
234 parameters
21 restraints
H atoms treated by a mixture of independent and constrained refinement
Δρ_max_ = 0.15 e Å^−3^
Δρ_min_ = −0.12 e Å^−3^

### 

Data collection: *SMART* (Bruker, 1997 ▶); cell refinement: *SAINT* (Bruker, 2003 ▶); data reduction: *SAINT*; program(s) used to solve structure: *SHELXS97* (Sheldrick, 2008 ▶); program(s) used to refine structure: *SHELXL97* (Sheldrick, 2008 ▶); molecular graphics: *X-SEED* (Barbour, 2001 ▶); software used to prepare material for publication: *publCIF* (Westrip, 2009 ▶).

## Supplementary Material

Crystal structure: contains datablocks global, I. DOI: 10.1107/S1600536809031225/hb5034sup1.cif

Structure factors: contains datablocks I. DOI: 10.1107/S1600536809031225/hb5034Isup2.hkl

Additional supplementary materials:  crystallographic information; 3D view; checkCIF report

## Figures and Tables

**Table 1:** Hydrogen-bond geometry (Å, °)

*D*—H⋯*A*	*D*—H	H⋯*A*	*D*⋯*A*	*D*—H⋯*A*
O1—H1⋯N2	0.85 (4)	2.00 (2)	2.831 (4)	164 (5)
O2—H2⋯N1	0.85 (4)	2.04 (2)	2.811 (4)	150 (4)

**Fig. 1.**
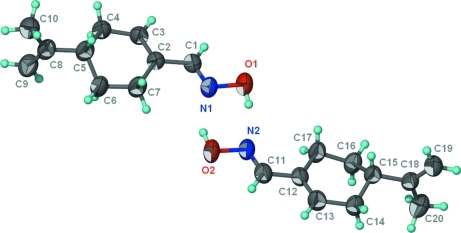
The molecular structure of (I) at the 50% probability level; hydrogen atoms are drawn as spheres of arbitrary radius. The disorder is not shown.

## supplementary crystallographic information

### Experimental

Hydroxylamine hydrochloride (3.15 g, 0.045 mol) in water (50 ml) was treated with sodium carbonate (2.12 g, 0.02 mol). To this solution was added perillaldehyde (4.50 g, 0.03 mol). The mixture was kept at 318 K for 2 h. The solution was cooled and the solid that formed was heated in water for another 2 h. This was repeated a second time. The product was recrystallized from ethyl acetate to yield colourless blocks of (I) (yield 5.5 g, 80%); m.p. 374–375 K.

### Refinement

Carbon-bound H-atoms were placed in calculated positions (C–H 0.95–0.97 Å) and were included in the refinement in the riding model approximation, with *U*(H) set to 1.2–1.5*U*(C).

The hydroxy H-atoms were located in a difference Fourier map, and were refined with a distance restraint of O–H 0.85±0.01 Å; their temperature factors were freely refined.

One of the propenyl groups is disordered over two positions; the disorder could not be refined, and was assumed to be a 1:1 type of disorder that involved only the terminal carbon atoms. The C–C single bond distance was restrained to 1.54±0.01 Å and the double bond distance to 1.35±0.01 Å. The displacement factors of the primed atoms were restrained to neqrly equal those of the umprimed ones, and the anisotropic displacement factors were restrained to be nearly isotropic.

Friedel pairs were merged. The configuration of the molecule was assumed to be that of the chiral starting reagent (*i.e.*, *S*-configuration).

### Crystal data

**Table:**

C_10_H_15_NO	*Z* = 2
*M**_r_* = 165.23	*F*(000) = 180
Triclinic, *P*1	*D*_x_ = 1.124 Mg m^−^^3^
Hall symbol: P 1	Mo *K*α radiation, λ = 0.71073 Å
*a* = 7.2679 (6) Å	Cell parameters from 1889 reflections
*b* = 8.1702 (7) Å	θ = 2.4–26.9°
*c* = 8.9426 (8) Å	µ = 0.07 mm^−^^1^
α = 105.150 (1)°	*T* = 293 K
β = 95.658 (1)°	Block, colorless
γ = 104.602 (1)°	0.48 × 0.42 × 0.22 mm
*V* = 488.25 (7) Å^3^	

### Data collection

**Table:**

Bruker SMART diffractometer	1457 reflections with *I* > 2σ(*I*)
Radiation source: fine-focus sealed tube	*R*_int_ = 0.014
graphite	θ_max_ = 27.1°, θ_min_ = 2.4°
φ and ω scans	*h* = −9→9
4074 measured reflections	*k* = −10→10
2078 independent reflections	*l* = −11→11

### Refinement

**Table:**

Refinement on *F*^2^	Primary atom site location: structure-invariant direct methods
Least-squares matrix: full	Secondary atom site location: difference Fourier map
*R*[*F*^2^ > 2σ(*F*^2^)] = 0.043	Hydrogen site location: inferred from neighbouring sites
*wR*(*F*^2^) = 0.139	H atoms treated by a mixture of independent and constrained refinement
*S* = 1.07	*w* = 1/[σ^2^(*F*_o_^2^) + (0.0793*P*)^2^ + 0.0172*P*] where *P* = (*F*_o_^2^ + 2*F*_c_^2^)/3
2078 reflections	(Δ/σ)_max_ < 0.001
234 parameters	Δρ_max_ = 0.15 e Å^−^^3^
21 restraints	Δρ_min_ = −0.12 e Å^−^^3^

### Fractional atomic coordinates and isotropic or equivalent isotropic displacement parameters (Å^2^)

**Table:**

	*x*	*y*	*z*	*U*_iso_*/*U*_eq_	Occ. (<1)
O1	0.5000 (4)	0.5002 (4)	0.5001 (3)	0.0885 (8)	
O2	0.7482 (4)	0.9157 (4)	0.6717 (4)	0.0922 (8)	
N1	0.4289 (4)	0.6405 (4)	0.4831 (3)	0.0724 (8)	
N2	0.8170 (4)	0.7770 (4)	0.6971 (3)	0.0727 (7)	
C1	0.2564 (5)	0.5857 (4)	0.4097 (4)	0.0699 (8)	
H1A	0.1932	0.4649	0.3775	0.084*	
C2	0.1551 (5)	0.7069 (4)	0.3745 (4)	0.0657 (8)	
C3	−0.0246 (5)	0.6464 (4)	0.2976 (4)	0.0758 (9)	
H3	−0.0836	0.5251	0.2706	0.091*	
C4	−0.1401 (5)	0.7574 (4)	0.2510 (4)	0.0776 (9)	
H4A	−0.2386	0.7643	0.3161	0.093*	
H4B	−0.2048	0.7010	0.1425	0.093*	
C5	−0.0167 (5)	0.9448 (4)	0.2672 (4)	0.0760 (9)	
H5	0.0572	0.9363	0.1809	0.091*	
C6	0.1263 (6)	1.0142 (4)	0.4182 (6)	0.0973 (13)	
H6A	0.0572	1.0176	0.5058	0.117*	
H6B	0.2013	1.1345	0.4291	0.117*	
C7	0.2618 (6)	0.9016 (5)	0.4248 (5)	0.0905 (12)	
H7A	0.3552	0.9237	0.3567	0.109*	
H7B	0.3320	0.9354	0.5314	0.109*	
C8	−0.1413 (5)	1.0638 (4)	0.2484 (4)	0.0784 (10)	0.50
C9	−0.2394 (18)	1.127 (2)	0.3557 (11)	0.088 (3)	0.50
H9A	−0.3134	1.1999	0.3366	0.105*	0.50
H9B	−0.2342	1.0994	0.4501	0.105*	0.50
C10	−0.1478 (19)	1.1072 (18)	0.0954 (12)	0.093 (3)	0.50
H10A	−0.2124	1.0017	0.0107	0.139*	0.50
H10B	−0.0186	1.1541	0.0797	0.139*	0.50
H10C	−0.2167	1.1936	0.0979	0.139*	0.50
C8'	−0.1413 (5)	1.0638 (4)	0.2484 (4)	0.0784 (10)	0.50
C9'	−0.1960 (17)	1.0794 (19)	0.1074 (12)	0.088 (3)	0.50
H9'A	−0.2772	1.1487	0.0964	0.105*	0.50
H9'B	−0.1529	1.0210	0.0200	0.105*	0.50
C10'	−0.208 (2)	1.157 (2)	0.3937 (11)	0.093 (3)	0.50
H10D	−0.2765	1.2350	0.3683	0.139*	0.50
H10E	−0.0981	1.2236	0.4747	0.139*	0.50
H10F	−0.2922	1.0702	0.4303	0.139*	0.50
C11	0.9893 (5)	0.8319 (4)	0.7688 (4)	0.0733 (9)	
H11	1.0553	0.9518	0.7953	0.088*	
C12	1.0866 (5)	0.7107 (4)	0.8108 (4)	0.0644 (8)	
C13	1.2696 (5)	0.7692 (4)	0.8782 (5)	0.0781 (10)	
H13	1.3307	0.8898	0.9017	0.094*	
C14	1.3864 (5)	0.6556 (4)	0.9198 (5)	0.0798 (10)	
H14A	1.4061	0.6763	1.0328	0.096*	
H14B	1.5121	0.6889	0.8899	0.096*	
C15	1.2887 (4)	0.4597 (4)	0.8383 (4)	0.0632 (7)	
H15	1.2949	0.4391	0.7262	0.076*	
C16	1.0754 (4)	0.4179 (4)	0.8527 (5)	0.0746 (9)	
H16A	1.0134	0.2921	0.8053	0.090*	
H16B	1.0640	0.4475	0.9631	0.090*	
C17	0.9742 (5)	0.5210 (4)	0.7724 (5)	0.0763 (10)	
H17A	0.9523	0.4671	0.6594	0.092*	
H17B	0.8492	0.5137	0.8041	0.092*	
C18	1.3935 (4)	0.3442 (4)	0.8964 (4)	0.0666 (8)	
C19	1.4889 (6)	0.2566 (5)	0.8038 (5)	0.0813 (9)	
H19A	1.5548	0.1866	0.8401	0.098*	
H19B	1.4904	0.2646	0.7021	0.098*	
C20	1.3869 (7)	0.3355 (6)	1.0597 (5)	0.0934 (11)	
H20A	1.4500	0.2509	1.0786	0.140*	
H20B	1.2548	0.2998	1.0733	0.140*	
H20C	1.4516	0.4500	1.1328	0.140*	
H1	0.599 (5)	0.568 (5)	0.568 (5)	0.115 (18)*	
H2	0.641 (4)	0.866 (5)	0.609 (5)	0.110 (17)*	

### Atomic displacement parameters (Å^2^)

**Table:**

	*U*^11^	*U*^22^	*U*^33^	*U*^12^	*U*^13^	*U*^23^
O1	0.096 (2)	0.0817 (17)	0.1005 (18)	0.0444 (17)	0.0085 (16)	0.0344 (14)
O2	0.095 (2)	0.0784 (16)	0.114 (2)	0.0444 (16)	0.0000 (17)	0.0349 (15)
N1	0.079 (2)	0.0743 (18)	0.0748 (16)	0.0356 (16)	0.0104 (15)	0.0285 (14)
N2	0.0716 (19)	0.0704 (17)	0.0855 (18)	0.0333 (15)	0.0092 (15)	0.0282 (14)
C1	0.078 (2)	0.0649 (19)	0.0689 (19)	0.0273 (18)	0.0089 (17)	0.0181 (15)
C2	0.068 (2)	0.0613 (18)	0.0666 (18)	0.0211 (15)	0.0059 (15)	0.0160 (14)
C3	0.071 (2)	0.0540 (18)	0.090 (2)	0.0132 (17)	−0.0072 (18)	0.0111 (16)
C4	0.065 (2)	0.0647 (19)	0.090 (2)	0.0149 (16)	−0.0079 (17)	0.0122 (17)
C5	0.0624 (19)	0.072 (2)	0.091 (2)	0.0177 (15)	0.0000 (16)	0.0272 (17)
C6	0.082 (2)	0.0569 (17)	0.134 (3)	0.0146 (16)	−0.035 (2)	0.0212 (19)
C7	0.071 (2)	0.068 (2)	0.119 (3)	0.0139 (18)	−0.021 (2)	0.024 (2)
C8	0.064 (2)	0.067 (2)	0.100 (3)	0.0135 (16)	−0.0078 (17)	0.0302 (18)
C9	0.097 (5)	0.108 (6)	0.076 (3)	0.048 (4)	0.020 (3)	0.040 (3)
C10	0.103 (5)	0.083 (4)	0.093 (4)	0.033 (4)	0.002 (3)	0.027 (3)
C8'	0.064 (2)	0.067 (2)	0.100 (3)	0.0135 (16)	−0.0078 (17)	0.0302 (18)
C9'	0.097 (5)	0.108 (6)	0.076 (3)	0.048 (4)	0.020 (3)	0.040 (3)
C10'	0.103 (5)	0.083 (4)	0.093 (4)	0.033 (4)	0.002 (3)	0.027 (3)
C11	0.075 (2)	0.0609 (19)	0.086 (2)	0.0230 (18)	0.0118 (18)	0.0229 (16)
C12	0.063 (2)	0.0578 (17)	0.0732 (18)	0.0205 (15)	0.0092 (15)	0.0195 (14)
C13	0.064 (2)	0.0537 (18)	0.107 (3)	0.0101 (16)	−0.0006 (18)	0.0200 (17)
C14	0.057 (2)	0.0608 (19)	0.112 (3)	0.0107 (16)	−0.0085 (18)	0.0231 (18)
C15	0.0576 (16)	0.0601 (16)	0.0705 (17)	0.0166 (13)	0.0037 (13)	0.0203 (13)
C16	0.0581 (18)	0.0659 (18)	0.100 (2)	0.0123 (15)	0.0015 (16)	0.0355 (18)
C17	0.056 (2)	0.066 (2)	0.105 (3)	0.0164 (16)	−0.0043 (17)	0.0292 (18)
C18	0.0528 (17)	0.0590 (18)	0.082 (2)	0.0124 (15)	−0.0022 (14)	0.0187 (15)
C19	0.080 (2)	0.0733 (19)	0.092 (2)	0.0299 (18)	0.0063 (18)	0.0227 (17)
C20	0.094 (3)	0.107 (3)	0.095 (2)	0.041 (2)	0.010 (2)	0.047 (2)

### Geometric parameters (Å, °)

**Table:**

O1—N1	1.407 (3)	C9'—H9'A	0.9300
O1—H1	0.85 (4)	C9'—H9'B	0.9300
O2—N2	1.408 (3)	C10'—H10D	0.9600
O2—H2	0.85 (4)	C10'—H10E	0.9600
N1—C1	1.266 (4)	C10'—H10F	0.9600
N2—C11	1.261 (5)	C11—C12	1.455 (4)
C1—C2	1.451 (4)	C11—H11	0.9300
C1—H1A	0.9300	C12—C13	1.316 (5)
C2—C3	1.320 (5)	C12—C17	1.489 (5)
C2—C7	1.506 (5)	C13—C14	1.495 (4)
C3—C4	1.487 (4)	C13—H13	0.9300
C3—H3	0.9300	C14—C15	1.520 (4)
C4—C5	1.529 (5)	C14—H14A	0.9700
C4—H4A	0.9700	C14—H14B	0.9700
C4—H4B	0.9700	C15—C18	1.511 (4)
C5—C6	1.500 (5)	C15—C16	1.527 (4)
C5—C8	1.514 (4)	C15—H15	0.9800
C5—H5	0.9800	C16—C17	1.514 (4)
C6—C7	1.515 (5)	C16—H16A	0.9700
C6—H6A	0.9700	C16—H16B	0.9700
C6—H6B	0.9700	C17—H17A	0.9700
C7—H7A	0.9700	C17—H17B	0.9700
C7—H7B	0.9700	C18—C19	1.316 (5)
C8—C9	1.327 (8)	C18—C20	1.485 (5)
C8—C10	1.500 (8)	C19—H19A	0.9300
C9—H9A	0.9300	C19—H19B	0.9300
C9—H9B	0.9300	C20—H20A	0.9600
C10—H10A	0.9600	C20—H20B	0.9600
C10—H10B	0.9600	C20—H20C	0.9600
C10—H10C	0.9600		
			
N1—O1—H1	94 (3)	H10E—C10'—H10F	109.5
N2—O2—H2	106 (3)	N2—C11—C12	121.0 (3)
C1—N1—O1	111.9 (3)	N2—C11—H11	119.5
C11—N2—O2	112.1 (3)	C12—C11—H11	119.5
N1—C1—C2	121.6 (3)	C13—C12—C11	120.0 (3)
N1—C1—H1A	119.2	C13—C12—C17	121.9 (3)
C2—C1—H1A	119.2	C11—C12—C17	118.0 (3)
C3—C2—C1	120.4 (3)	C12—C13—C14	124.6 (3)
C3—C2—C7	121.3 (3)	C12—C13—H13	117.7
C1—C2—C7	118.3 (3)	C14—C13—H13	117.7
C2—C3—C4	125.1 (3)	C13—C14—C15	112.1 (3)
C2—C3—H3	117.5	C13—C14—H14A	109.2
C4—C3—H3	117.5	C15—C14—H14A	109.2
C3—C4—C5	112.7 (3)	C13—C14—H14B	109.2
C3—C4—H4A	109.1	C15—C14—H14B	109.2
C5—C4—H4A	109.1	H14A—C14—H14B	107.9
C3—C4—H4B	109.1	C18—C15—C14	112.0 (2)
C5—C4—H4B	109.1	C18—C15—C16	114.2 (2)
H4A—C4—H4B	107.8	C14—C15—C16	108.9 (3)
C6—C5—C8	114.0 (3)	C18—C15—H15	107.1
C6—C5—C4	109.6 (3)	C14—C15—H15	107.1
C8—C5—C4	111.3 (3)	C16—C15—H15	107.1
C6—C5—H5	107.2	C17—C16—C15	111.1 (2)
C8—C5—H5	107.2	C17—C16—H16A	109.4
C4—C5—H5	107.2	C15—C16—H16A	109.4
C5—C6—C7	112.3 (3)	C17—C16—H16B	109.4
C5—C6—H6A	109.2	C15—C16—H16B	109.4
C7—C6—H6A	109.2	H16A—C16—H16B	108.0
C5—C6—H6B	109.2	C12—C17—C16	112.7 (3)
C7—C6—H6B	109.2	C12—C17—H17A	109.0
H6A—C6—H6B	107.9	C16—C17—H17A	109.0
C2—C7—C6	111.9 (3)	C12—C17—H17B	109.0
C2—C7—H7A	109.2	C16—C17—H17B	109.0
C6—C7—H7A	109.2	H17A—C17—H17B	107.8
C2—C7—H7B	109.2	C19—C18—C20	122.0 (3)
C6—C7—H7B	109.2	C19—C18—C15	120.0 (3)
H7A—C7—H7B	107.9	C20—C18—C15	118.0 (3)
C9—C8—C10	120.8 (8)	C18—C19—H19A	120.0
C9—C8—C5	124.4 (6)	C18—C19—H19B	120.0
C10—C8—C5	114.7 (6)	H19A—C19—H19B	120.0
C8—C9—H9A	120.0	C18—C20—H20A	109.5
C8—C9—H9B	120.0	C18—C20—H20B	109.5
H9A—C9—H9B	120.0	H20A—C20—H20B	109.5
H9'A—C9'—H9'B	120.0	C18—C20—H20C	109.5
H10D—C10'—H10E	109.5	H20A—C20—H20C	109.5
H10D—C10'—H10F	109.5	H20B—C20—H20C	109.5
			
O1—N1—C1—C2	−178.2 (3)	O2—N2—C11—C12	178.3 (3)
N1—C1—C2—C3	179.9 (3)	N2—C11—C12—C13	176.4 (3)
N1—C1—C2—C7	1.2 (5)	N2—C11—C12—C17	−2.5 (5)
C1—C2—C3—C4	−178.0 (3)	C11—C12—C13—C14	−177.1 (3)
C7—C2—C3—C4	0.7 (6)	C17—C12—C13—C14	1.7 (6)
C2—C3—C4—C5	13.1 (5)	C12—C13—C14—C15	15.1 (6)
C3—C4—C5—C6	−42.4 (4)	C13—C14—C15—C18	−172.6 (3)
C3—C4—C5—C8	−169.4 (3)	C13—C14—C15—C16	−45.3 (4)
C8—C5—C6—C7	−174.0 (4)	C18—C15—C16—C17	−172.3 (3)
C4—C5—C6—C7	60.5 (5)	C14—C15—C16—C17	61.8 (4)
C3—C2—C7—C6	15.8 (5)	C13—C12—C17—C16	13.8 (5)
C1—C2—C7—C6	−165.5 (3)	C11—C12—C17—C16	−167.3 (3)
C5—C6—C7—C2	−46.7 (5)	C15—C16—C17—C12	−45.5 (4)
C6—C5—C8—C9	−51.0 (9)	C14—C15—C18—C19	−109.8 (4)
C4—C5—C8—C9	73.5 (8)	C16—C15—C18—C19	125.9 (3)
C6—C5—C8—C10	129.0 (7)	C14—C15—C18—C20	69.4 (4)
C4—C5—C8—C10	−106.4 (7)	C16—C15—C18—C20	−54.9 (4)

### Hydrogen-bond geometry (Å, °)

**Table:**

*D*—H···*A*	*D*—H	H···*A*	*D*···*A*	*D*—H···*A*
O1—H1···N2	0.85 (4)	2.00 (2)	2.831 (4)	164 (5)
O2—H2···N1	0.85 (4)	2.04 (2)	2.811 (4)	150 (4)
